# Facile Synthesis of Radial-Like Macroporous Superparamagnetic Chitosan Spheres with In-Situ Co-Precipitation and Gelation of Ferro-Gels

**DOI:** 10.1371/journal.pone.0049329

**Published:** 2012-11-30

**Authors:** Chih-Hui Yang, Chih-Yu Wang, Keng-Shiang Huang, Chen-Sheng Yeh, Andrew H. -J. Wang, Wei-Ting Wang, Ming-Yu Lin

**Affiliations:** 1 Department of Biological Science & Technology, I-Shou University, Kaohsiung, Taiwan; 2 Department of Biomedical Engineering, I-Shou University, Kaohsiung, Taiwan; 3 The School of Chinese Medicine for Post-Baccalaureate, I-Shou University, Kaohsiung, Taiwan; 4 Department of Chemistry, National Cheng-Kung University, Tainan, Taiwan; 5 Institute of Biological Chemistry, Academia Sinica, Taipei, Taiwan; 6 Instrument Technology Research Center, National Applied Research Laboratories, Taiwan; 7 Institute of Molecular Medicine and Bioengineering, National Chiao Tung University, Hsinchu, Taiwan; Harbin Institute of Technology, China

## Abstract

Macroporous chitosan spheres encapsulating superparamagnetic iron oxide nanoparticles were synthesized by a facile and effective one-step fabrication process. Ferro-gels containing ferrous cations, ferric cations and chitosan were dropped into a sodium hydroxide solution through a syringe pump. In addition, a sodium hydroxide solution was employed for both gelation (chitosan) and co-precipitation (ferrous cations and ferric cations) of the ferro-gels. The results showed that the in-situ co-precipitation of ferro-ions gave rise to a radial morphology with non-spheroid macro pores (large cavities) inside the chitosan spheres. The particle size of iron oxide can be adjusted from 2.5 nm to 5.4 nm by tuning the concentration of the sodium hydroxide solution. Using Fourier Transform Infrared Spectroscopy and X-ray diffraction spectra, the synthesized nanoparticles were illustrated as Fe_3_O_4_ nanoparticles. In addition, the prepared macroporous chitosan spheres presented a super-paramagnetic behaviour at room temperature with a saturation magnetization value as high as ca. 18 emu/g. The cytotoxicity was estimated using cell viability by incubating doses (0∼1000 µg/mL) of the macroporous chitosan spheres. The result showed good viability (above 80%) with alginate chitosan particles below 1000 µg/mL, indicating that macroporous chitosan spheres were potentially useful for biomedical applications in the future.

## Introduction

Porous spheres have several, extremely valuable therapeutic and biotechnological applications [1], [2], including cell immobilization, drug delivery, and as a packing material in chromatography [3]–[6]. Macroporous structures are especially important to spheres to improve their performance [1], [2], [7]. For example, large pores can increase the drug permeability of the spheres in drug delivery, significantly increase their specific surface area, allow them to be used as culture systems for growing adherent cells, be used as water remediation in high diffusion rates, or be used in the separation of large biomolecules, etc [8]–[16].

Chitosan itself is eco-friendly due to the properties of non-toxic, biodegradable and bio-compatible, and has wide applications on medicine, pharmacy, and environmental protection [17]–[19]. Highly porous chitosan beads or scaffolds are especially useful for bone tissue engineering, drug delivery, and heavy metal adsorption [20]–[22]. Furthermore, macroporous chitosan structure has special advantages in some applications [23]–[25]. For example, Xi and Wu employed macroporous chitosan-coated silica gel to accommodate the accessibility of the protein adsorption [23]. Wu *et al.* employed macroporous chitosan-silica gel beads for the immobilized affinity chromatographic (IMAC) absorbents [24]. Li *et al.* fabricated spherical chitosan with macro reticular structure on removal of heavy metals from wastewaters [25]. In addition, incorporating iron oxide nanoparticles and macroporous chitosan matrices exhibits specific mechanical and functional properties, that can lead to wide variety of applications, such as recyclable heavy metal removal, magnetic-induced tumor therapy, responsive drug release, magnetic resonance imaging (MRI) enhancement, etc [26]–[28].

The preparation of chitosan particles with/without iron oxide nanoparticles have been investigated in the literatures, such as emulsification, ionotropic gelation, reverse micellar, solvent evaporation, spray drying, freeze-drying, coacervation, sieving, porogen leaching out, alkaline gelation, and other techniques [17]–[32]. However, most of the pores in these above mentioned studies were spherical. To the best of our knowledge, the synthesis of a patterned, macroporous structure (e.g. radial-like and interconnected large cavities) has rarely been discussed in the literature.

**Figure 1:**
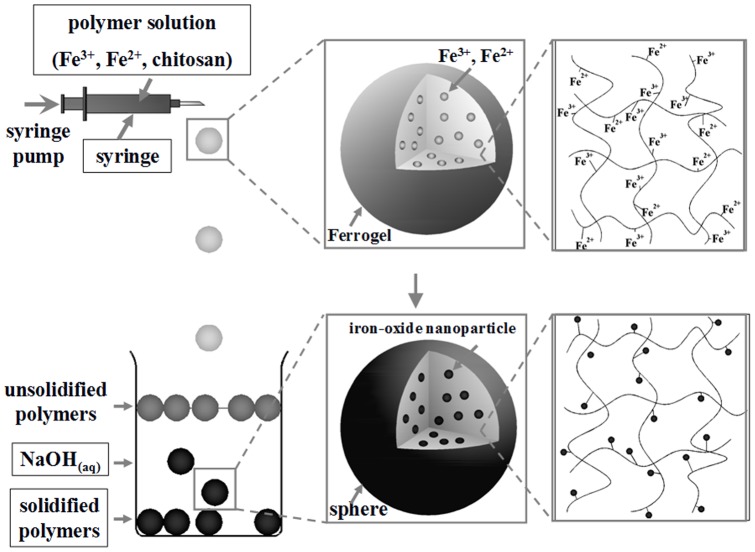
Schematic of the formation process of macroporous chitosan spheres.

Herein we reported an approach for novel macroporous chitosan spheres with a non-spheroid, patterned (*i.e.* radial-like) morphology. **Figure 1** shows the one step process for macroporous chitosan spheres production through *in-situ* co-precipitation and the gelation of ferro-gels. Due to the abundant amino groups, chitosan has a good capability for the uptake of ferrous and ferric cations *via* the chelating or ion exchange mechanisms [33]. Ferrous and ferric cations were mixed with a chitosan solution to form the ferro-gel solution. Subsequently the ferro-gel was dropped into a NaOH solution for simultaneous chitosan solidification and iron cations co-precipitation and form the iron oxide nanoparticles embedded chitosan spheres. The color of the solution changed quickly from yellow-orange to black, indicating that iron oxide nanoparticles are formed through the co-precipitation reaction [34]. The strategy of the iron oxide nanoparticles synthesis is based on a ferro-solution containing Fe^2+^ and Fe^3+^ dissolved in 1∶2 molar ratios.

## Experimental Section

### Materials

Chitosan (molecular weight: 150,000), iron(II) chloride tetrahydrate (FeCl_2_•4H_2_O, 99%), iron (III) chloride hexahydrate (FeCl_3_•6H_2_O, 98%), and sodium hydroxide (NaOH) were purchased from SIGMA, J. T. Baker, Alfa Aesar and Mallinckrodt, respectively, and used as received without further processing.

### Synthesis of the chitosan spheres

Chitosan (0.1 g, dissolved in 2 mL of 1%, v/v CH_3_COOH solution) were prepared and dropped into NaOH solutions of various concentrations (5, 20, 30, and 50 wt %) respectively, by means of a syringe and pump. After 10 minutes, chitosan spheres were observed. Spheres were collected by centrifugation, and were washed twice with 30 mL dd-H_2_O to remove any alkali. The spheres were then dried (EYELA FDU-1100) in vacuum at −54°C for 24 hours. The dried particles were stored at 4°C until use.

### Synthesis of the Fe_3_O_4_-chitosan composite spheres

Chitosan (0.1 g, dissolved in 2 mL of 1%, v/v CH_3_COOH solution), FeCl_2_•4H_2_O (0.0224 g, dissolved in 0.5 mL of 2N HCl solution) and FeCl_3_•6H_2_O (0.0448 g, dissolved in 0.5 mL of 2N HCl solution) were mixed through constant stirring for 30 minutes, until a ferro-gel solution was obtained. The ferro-gel solution was then dropped into NaOH solutions of various concentrations (5, 20, 30, and 50 wt %) respectively, by means of a syringe and pump. After 10 minutes, Fe_3_O_4_-chitosan composite spheres having a black color were observed. Spheres were collected by centrifugation, and were washed twice with 30 mL dd-H_2_O to remove any alkali. The spheres were then dried (EYELA FDU-1100) in vacuum at −54°C for 24 hours. The dried particles were stored at 4°C until use.

### Characterization

Size distributions of the various droplets samples were obtained from the random sampling of about 50 individual spheres so as to minimize selection bias. An inverted microscope system, including an optical microscope (BX60, Olympus, Japan) and a digital camera (DP70, Olympus, Japan), were employed for imaging. The average diameter of the spheres, expressed as mean ± standard deviation, was obtained from the photomicrograph. Statistical analysis was performed on the size distribution using an unpaired t-test. X-ray diffraction (XRD, D8 Advance, PANalytical X'PERT PRO) patterns were obtained at room temperature by using Cu K-α radiation (λ = 1.5406 Å) with a range of 2θ = 20°∼80°, and a scanning rate of 0.05 s^−1^. Fourier transform infrared spectroscopy (FTIR) spectra were recorded with a Spectrum RXI FTIR Spectrometer, using KBr pellets, in the range of 400∼4000 cm^−1^, with a resolution of 4 cm^−1^. The morphology of the composites was analyzed using a scanning electron microscope (SEM, Hitachi, S-2700, Japan), and a transmission electron microscope (TEM, FEI Tecnai G2 20 S-Twin, EDS: METEK). The magnetic properties were measured with a superconducting quantum interference device (SQUID, MPMS-XL7) and were evaluated in terms of saturation magnetization and coercivity. The lyophilized microcapsules were placed in a capsule and wrapped with Teflon to prevent leakage. The magnetic susceptibility was measured with a magnetic field of 60 KOe at 300 K. The zero-field-cooled (ZFC) and field-cooled (FC) magnetization curves were measured at a temperature ranging between 5 and 300 K at a 500 Orested applied field.

### Cell viability analysis

The viability of the control and the treated cells were evaluated using an MTT (3-(4,5-cimethylthiazol-2-yl)-2,5-diphenyl tetrazolium bromide) assay with human glioblastoma U87MG, human breast adenocarcinoma MCF-7, and glioblastoma multiforme (GBM) cells lines. Cells (1×104/well) were seeded in 96-well microtiter plates containing 100 µL culture medium, permitted to adhere for 18 hours, washed with phosphate buffered saline, and then treated with various Fe_3_O_4_-chitosan composite spheres. After 24 hours of treatment, cells were incubated at 37°C in 200 µL MTT solution (1 mg/mL) for 4 hours. After removal of the medium and MTT, 100 µL dimethyl sulfoxide (DMSO) was added to each well, and the assay plate was read at 595 nm using a microplate reader (Thermo ELECTRON CORPORATION MULTISKAN ASCENT). The absorbance of untreated cells was considered as 100%.

## Results and Discussion

**Figure 2:**
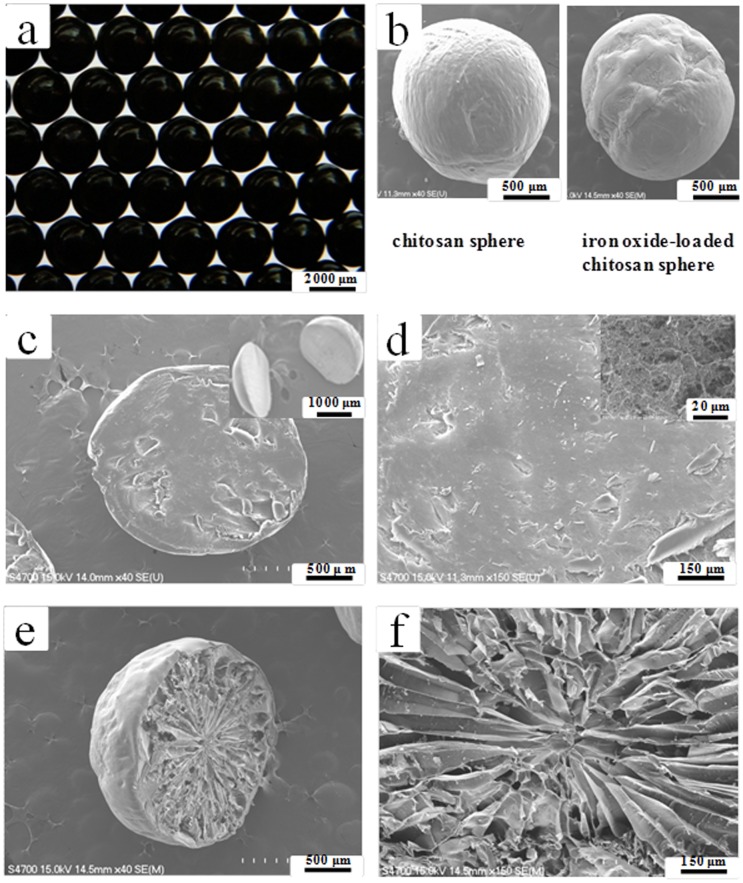
Morphologies of the synthesyzed chitosan spheres. (a) An optical image of the iron oxide nanoparticles loaded chitosan spheres. (b) SEM images of chitosan spheres without (left) and with (right) iron oxide nanoparticles. (c) and (d) show the cross-section images of chitosan spheres (without iron oxide nanoparticles). Inset in (c) shows the sliced hemispheres of chitosan sphere. Inset in (d) shows details of the internal structure. (e) and (f) show the cross-section images of iron oxide nanoparticles loaded chitosan spheres.

**Figure 2a** shows a photograph of the prepared iron oxide-loaded chitosan spheres. It is evident that the spheres are uniform in size, measuring 2.67±0.08 mm in diameter. After the drying process, the spheres had shrunk by about 79%. The diameter of the spheres could be controlled from 1 to 5 mm (with a variation of less than 8%) by altering the size of the needles under the relative sample flow rate from 20 to 30 mL/hour. In the future, the diameter of the spheres could be made smaller by employing other conventional droplet generation methods, such as atomization (spraying), emulsification, coacervation, sonication, electrostatic droplets, microfluidic droplets, etc [35]–[38]. It is worth noting that the surface of the iron oxide-loaded chitosan sphere is more irregular than that of pure chitosan spheres (**Fig. 2b**). This finding gave us the motivation to explore the internal structure of the polymer spheres. The pure chitosan sphere (absence of iron oxide nanoparticles) possesses a compact intra structure (**Figs. 2c and 2d**). In contrast, to our surprise, the inner structure of the iron oxide-loaded chitosan sphere shows a radial-patterned-like, non-spheroid morphology of macro pores and interconnected large cavities, as shown in **Figs. 2e and 2f**.

We presume that this special pattern of intra macroporous structures is due to the interaction of iron-oxide co-precipitation and chitosan gelation. Similar results were obtained by using gold ions for the synthesis of chitosan particles. A cellular-patterned-like cross-section structure and a micro-channeled-like longitudinal structure were achieved through the combination of the ISISA (ice-segregation-induced self-assembly) process and co-precipitation of gold nanoparticles [39]. Hortigüela *et al.* demonstrated that macro pores size and shape differed depending on the HAuCl_4_•3H_2_O concentration in the starting solution. In addition, the ISISA is related to the viscosity of the solution and eventually to the hydrogel strength. Deville *et al.* demonstrated that ice formation could be used to develop sophisticated porous polymer scaffolds [40]. In addition, Zhang *et al.* further showed that aligned porous polymer-inorganic composite materials by templating the polymer structure by means of instability formation whilst simultaneously freeze concentrating inorganic nanoparticles [41]. In our experiments, however, the only factor that caused the formation of macroporous structure is the *in situ* generated iron oxide nanoparticles (please see **Figs. 2c** vs. **2e**). Without the process of iron oxide nanoparticles formation, the macroporous structure is absent (**Fig. 2c**). To understand the exact mechanism, however, requires further investigation. The proposed route for the generation of chitosan spheres with non-spheroid macro pores is quite novel compared with the other synthetic methods [42]–[45].

Furthermore, the diameter of the iron oxide nanoparticles could be tuned by adjusting the concentration of the NaOH solution. The diameter of the prepared iron oxide spheres were identified by a transmission electron microscope (TEM). The particle size of iron oxide decreases when the NaOH concentration increases, e.g. the mean diameters were 5.4±1.7 nanometers (with 5% NaOH), 4.4±0.9 nanometers (with 20% NaOH), 3.8±0.9 nanometers (with 30% NaOH), and 2.5±0.2 nanometers (with 50% NaOH), respectively (**Figs. S1a**∼**S1d**). At the same time, radial macroporous structures were observed under all of the above conditions. In addition, the effects of chitosan concentration and molecular weight on the size of iron-oxide nanoparticles were studied. There was no significant difference among these conditions. The prepared iron oxide particles were in the range of 2.5 nm to 7 nm (**Fig. S2**). Finally, we will briefly describe the main findings and contributions of this study: (i) a facile and one-step method for fabricating macroporous chitosan spheres was established; (ii) radiating cavities and a non-spheroid structure of macroporous chitosan spheres was obtained; and (iii) size-adjustable iron oxide nanoparticles were synthesized and embedded in the chitosan spheres simultaneously.

**Figure 3:**
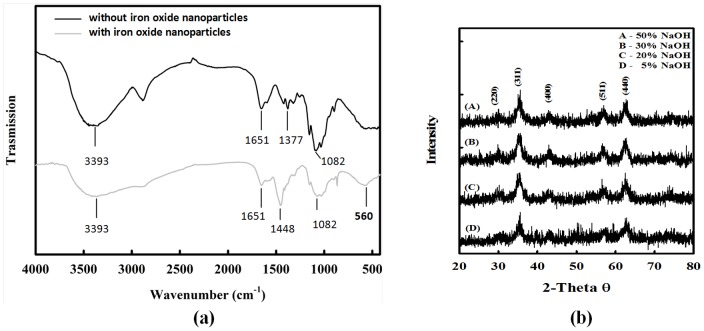
Characterization of the iron oxide-chitosan composite spheres. (a) FTIR spectra of chitosan spheres (gray line) and iron oxide nanoparticle-loaded chitosan spheres (black line). (b) X-ray diffraction pattern from iron oxides nanoparticle-loaded chitosan spheres prepared from the NaOH solutions with various concentrations.

The properties of prepared iron oxide nanoparticles-loaded chitosan spheres were characterized. The fact that these spheres contained iron oxide nanoparticles was determined by an atomic absorption spectrophotometer (AAS). The results show that each chitosan sphere (2.67±0.08 mm in diameter, 5 wt % NaOH solution) contained on average 125.95 mg/mL of ferro ions (e.g. each chitosan sphere contained 1.4 mg of iron oxide nanoparticles). **Figure 3a** shows the FTIR spectra of chitosan spheres and iron oxide nanoparticle-loaded chitosan spheres (produced from a 5 wt % NaOH solution). Characteristic peaks of chitosan spheres were evident in spectrum at 3393 cm^−1^ (-OH group), 1651 cm^−1^ (N-H bending vibration), and 1377 cm^−1^ (-C-O stretching of primary alcoholic group), respectively. The characteristic absorption peak for iron oxide nanoparticles was observed at ∼560 cm^−1^ (Fe-O group, iron oxide nanoparticle-loaded chitosan spheres). Similar results were obtained in other researches [26]–[29]. Therefore, it was concluded that the embedded nanoparticles were iron oxide nanoparticles. **Figure 3b** shows X-ray diffraction (XRD) spectra, which revealed that the synthesized nanoparticles were Fe_3_O_4_ phase, since the position and relative intensity of all diffraction peaks of samples were consistent with the crystalline pattern of Fe_3_O_4_ phase [29]. In addition, the chitosan joining the synthesis process did not cause any phase changes in the Fe_3_O_4_ nanoparticles. The shape of each peak was broadened due to the influence of chitosan polymer [29], [46]–[47].

The abovementioned results suggest that chitosan spheres have a superparamagnetic property, because of the Fe_3_O_4_ nanoparticles. The photograph in **Fig. S3** shows that Fe_3_O_4_ nanoparticles-loaded chitosan spheres can be attracted and drawn through the solution to the side wall of the vial by an external magnetic field. The magnetic properties were studied using a superconducting quantum interference device (SQUID). The plots of typical magnetization loops of magnetization (*M*) versus the magnetic field (*H*) at 300 K are shown in **Fig. S4a**. The saturation magnetization (M_s_), remanent magnetization (*M_r_*), coercivity (*H_c_*) and squareness (*S_r_* = *M_r_*/*M_s_*) were calculated from the plot. Since no remanence and coercivity was observed, it was concluded that the Fe_3_O_4_ nanoparticles-loaded chitosan spheres possess superparamagnetic nature at room temperature [48]. From the *M*–*H* loops, the saturation magnetization (*Ms*) was determined to be 18, 17, 15, and 14 emu/g, for the samples prepared from 5, 20, 30, and 50 wt % NaOH solutions, respectively. The saturation magnetization decreased when the NaOH concentration increased. This phenomenon can be explained by the fact that the magnetization of small iron oxide particles decreases as the particle size decreases [49]. Field-cooled (FC) and zero-field-cooled (ZFC) magnetization curves were taken under an external magnetic field of 500 Orested. **Figure S4b** shows the FC and ZFC curves for the samples prepared from 5, 20, 30, and 50 wt % NaOH solutions, respectively, ranging from 5 to 300 K. The ZFC and FC curves coincided above 40 K and separated below 40 K. The ZFC curve shows a broad peak at T_max_ 30±10 K indicative of a characteristic blocking temperature for superparamagnetic particles [50].

Stability of the beads in 37°C water (pH = 7) was tested for three days (72 hours). The experimental setup was shown in **Fig. S5a**. **Figure S5b** showed the beads (made from 5%, 20%, 30% and 50% NaOH solution, respectively) placed in the 37°C water at the beginning. **Figure S5c** showed the same beads after 3 days. Result suggests that the beads did not swell nor dissolve in 37°C water for at least 3 days. This suggests that the beads were stable in aqueous condition for at least 72 hours. In addition, stability in acidic condition was also tested by placing beads in 1% acetic acid (at room temperature). Chitosan beads start to dissolve in 20 minutes (**Fig. S6a**). The beads made from 5% and 20% NaOH were almost dissolved after 3.5 hours (**Fig. S6b**). All the beads made from various concentration were dissolved after 6 hours (**Fig. S6c**). Result shows that the beads were not suitable to be preserved in acidic condition.

The nanoparticle-loaded chitosan spheres were evaluated for pernicious biological properties by MTT assay. The cytotoxicity was estimated using cell viability by incubating doses (0∼1000 µg/mL) of the microparticles with U87MG (BCRC ID: 60360), MCF-7 (BCRC ID: 60436), and GBM (BCRC ID: 60164) cells for 24 hours (**Fig. S7**). All the cells lines were purchased from Biosource Center and Research Center, Food Insdustry Research and Development Institite, Taiwan. The results showed good viability (above 80%) of a series of Fe_3_O_4_-chitosan composite containing up to 1000 µg/mL of micro particles, indicating that the spheres are biologically friendly and can potentially be used in biological and biomedical applications in the future.

## Conclusions

Chitosan spheres with non-spheroid, radial-like macro pores were successfully obtained. By employing the *in-situ* co-precipitation for ferro-gels, the macro pores with radiating cavities were observed. The proposed method provides an alternative way for generating macroporous spheres. In addition, a sodium hydroxide solution was employed for both the gelation (chitosan) and co-precipitation (ferrous cations and ferric cations) of ferro-gels. The results show that the diameter of both the iron oxide nanoparticles and the chitosan were controllable. In addition, the prepared iron oxide nanoparticles loaded chitosan spheres present a superparamagnetic character with good viability (above 80% for chitosan spheres containing iron-oxide up to 1000 µg/mL), indicating that they are eco-friendly and can potentially be used in various biological and biomedical applications, such as magnetic-responsive drug delivery systems, scaffold for bone tissues, ultrasound contrast agent, targeted drug delivery vehicle, and for delivering tumor and/or thrombus destruction materials.

## Supporting Information

Figure S1 TEM images of iron oxide nanoparticles-loaded chitosan spheres prepared in various concentrations of NaOH solutions with (a) 5%, (b) 20%, (c) 30%, and (d) 50%, respectively.
(TIF)

Figure S2 The effects among chitosan concentration, chitosan molecular weight on the size of magnetic iron-oxide (MIO) nanoparticles.
(TIF)

Figure S3 Iron oxide nanoparticles-loaded chitosan spheres were attracted to wall of the vial by using an external magnetic field.
(TIF)

Figure S4 Magnetic characteristics of the iron oxide nanoparticles-loaded chitosan spheres. (a) Magnetization plots as a function of the applied field at 300 K. (b) Temperature dependent ZFC-FC magnetization curves measured at 500 Oe.
(TIF)

Figure S5 Stability test of the spheres in 37°C water (pH = 7). (a) shows the experimental setup. Spheres (made from 5%, 20%, 30% and 50% NaOH solution, respectively) deposed in the 37°C water (b) at the beginning, and (c) for 3 days.
(TIF)

Figure S6 Spheres placed in 1% acetic acid for (a) 20 minutes; (b) 3.5 hours; and (c) 6 hours.
(TIF)

Figure S7 Pernicious biological properties test for the nanoparticle-loaded chitosan spheres. (a) The cytotoxicity of iron oxide nanoparticles-loaded chitosan spheres (5.4±1.7 nanometers of iron oxide nanoparticles (with 5% NaOH) inside chitosan spheres) with three cell lines. (b) The MTT assay data of various iron oxide nanoparticles loaded chitosan spheres (MCF-7 cells). Sample A: 5.4±1.7 nanometers of iron oxide nanoparticles (with 5% NaOH) inside chitosan spheres. Sample B: 4.4±0.9 nanometers of iron oxide nanoparticles (with 20% NaOH) inside chitosan spheres. Sample C: 3.8±0.9 nanometers of iron oxide nanoparticles (with 30% NaOH) inside chitosan spheres. Sample D: 2.5±0.2 nanometers of iron oxide nanoparticles (with 50% NaOH) inside chitosan spheres.
(TIF)
