# Supplementary figures and images for: Facile Synthesis of Radial-Like Macroporous Superparamagnetic Chitosan Spheres with In-Situ Co-Precipitation and Gelation of Ferro-Gels
Source: PLoS One. 2012 Nov 30;7(11):e49329. doi: 10.1371/journal.pone.0049329 (PMC3511509; doi:10.1371/journal.pone.0049329)

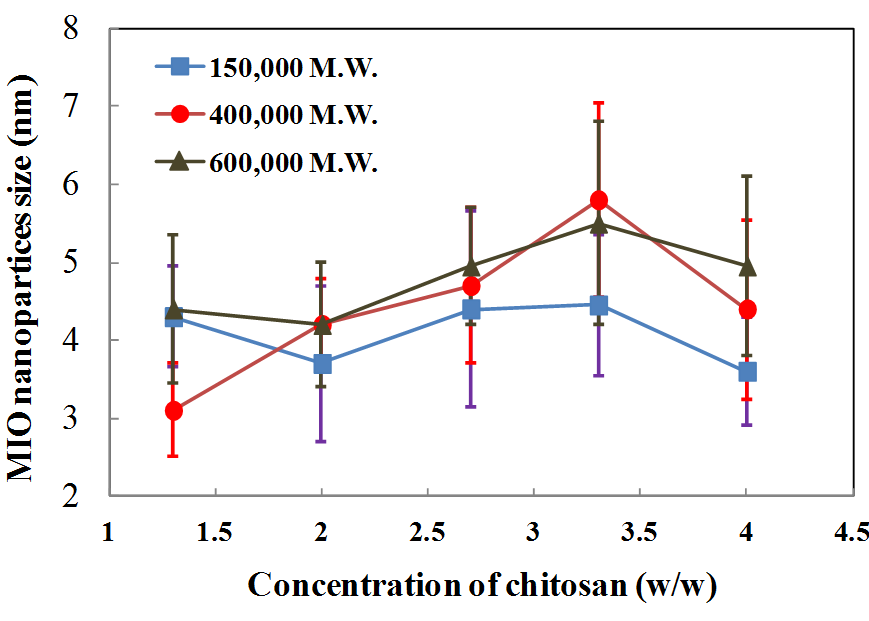

Supplement: Figure S2 — The effects among chitosan concentration, chitosan molecular weight on the size of magnetic iron-oxide (MIO) nanoparticles. (TIF) [file pone.0049329.s002.tif]

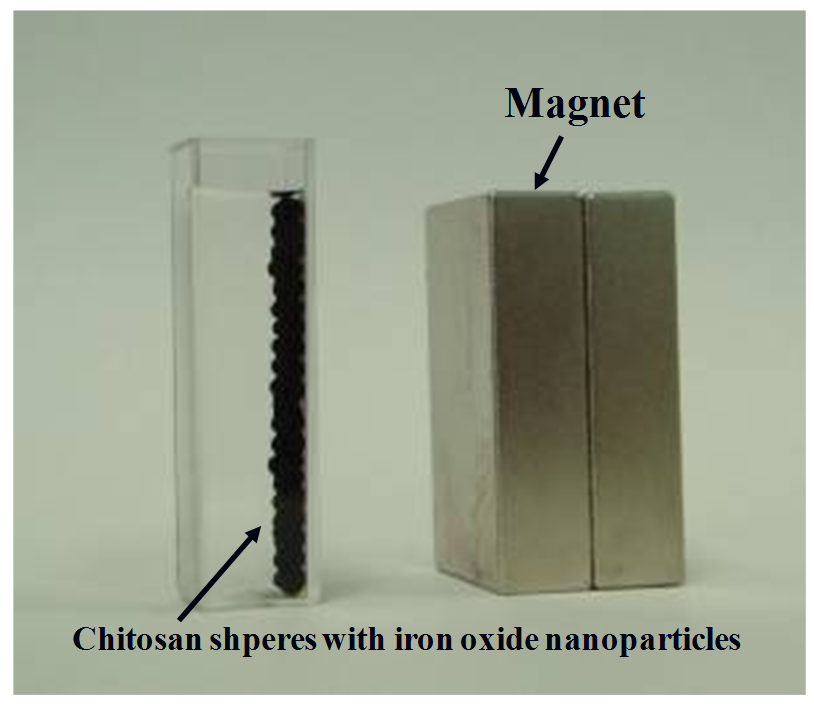

Supplement: Figure S3 — Iron oxide nanoparticles-loaded chitosan spheres were attracted to wall of the vial by using an external magnetic field. (TIF) [file pone.0049329.s003.tif]

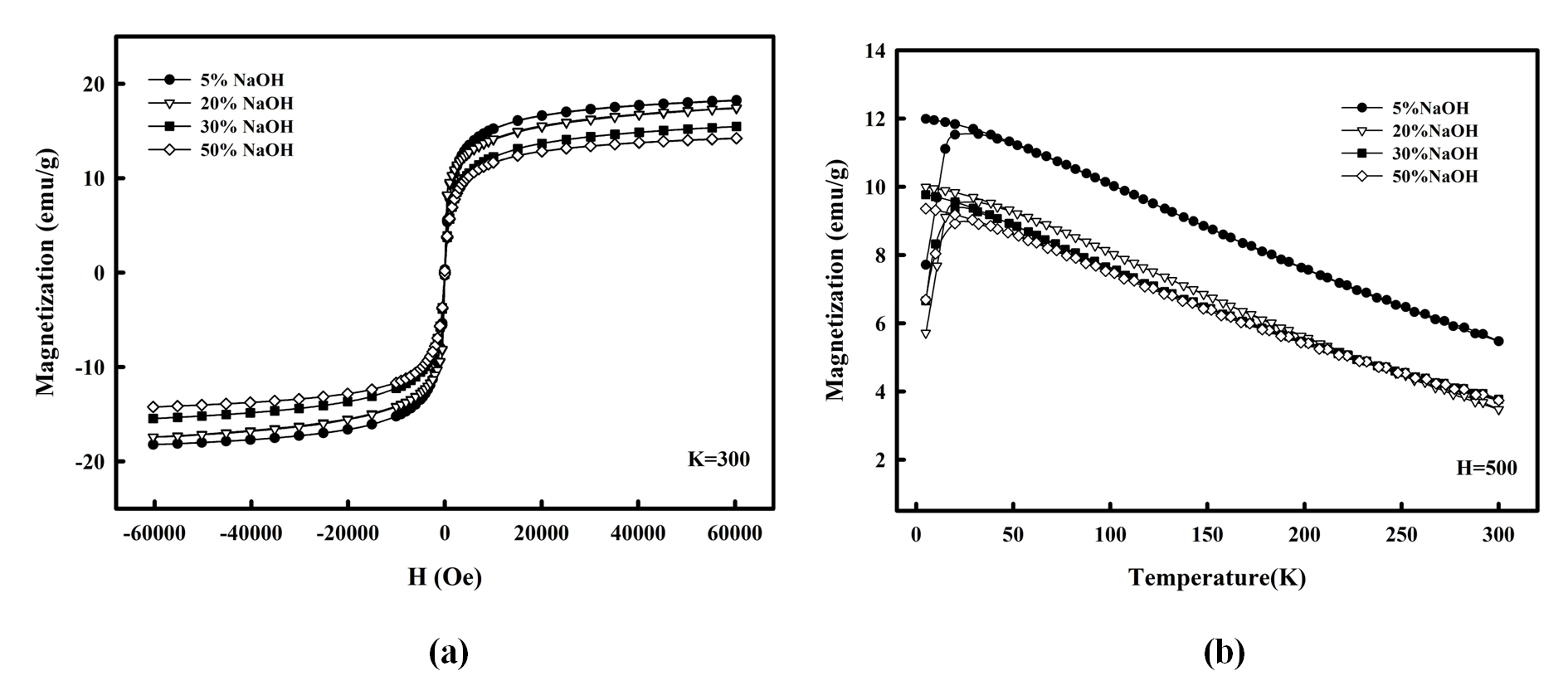

Supplement: Figure S4 — Magnetic characteristics of the iron oxide nanoparticles-loaded chitosan spheres. (a) Magnetization plots as a function of the applied field at 300 K. (b) Temperature dependent ZFC-FC magnetization curves measured at 500 Oe. (TIF) [file pone.0049329.s004.tif]

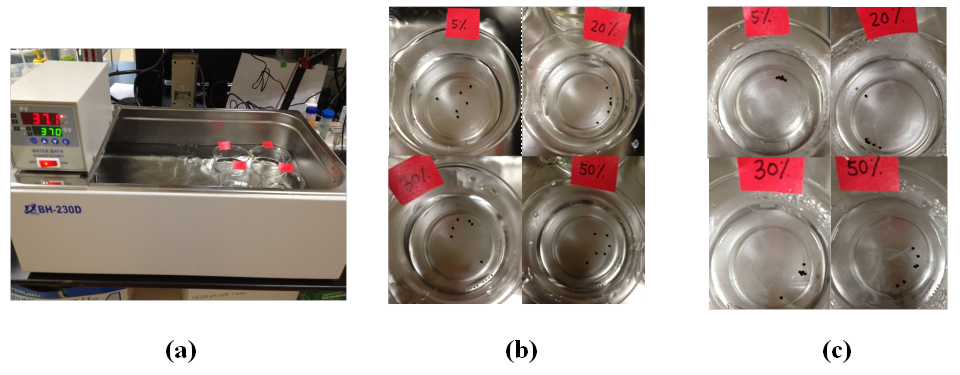

Supplement: Figure S5 — Stability test of the spheres in 37°C water (pH = 7). (a) shows the experimental setup. Spheres (made from 5%, 20%, 30% and 50% NaOH solution, respectively) deposed in the 37°C water (b) at the beginning, and (c) for 3 days. (TIF) [file pone.0049329.s005.tif]

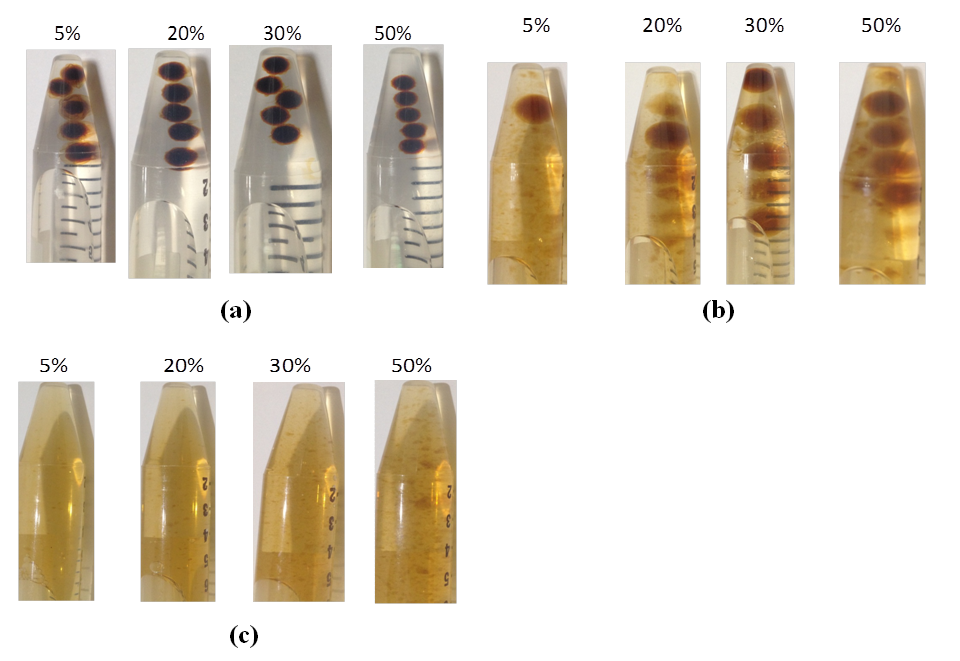

Supplement: Figure S6 — Spheres placed in 1% acetic acid for (a) 20 minutes; (b) 3.5 hours; and (c) 6 hours. (TIF) [file pone.0049329.s006.tif]

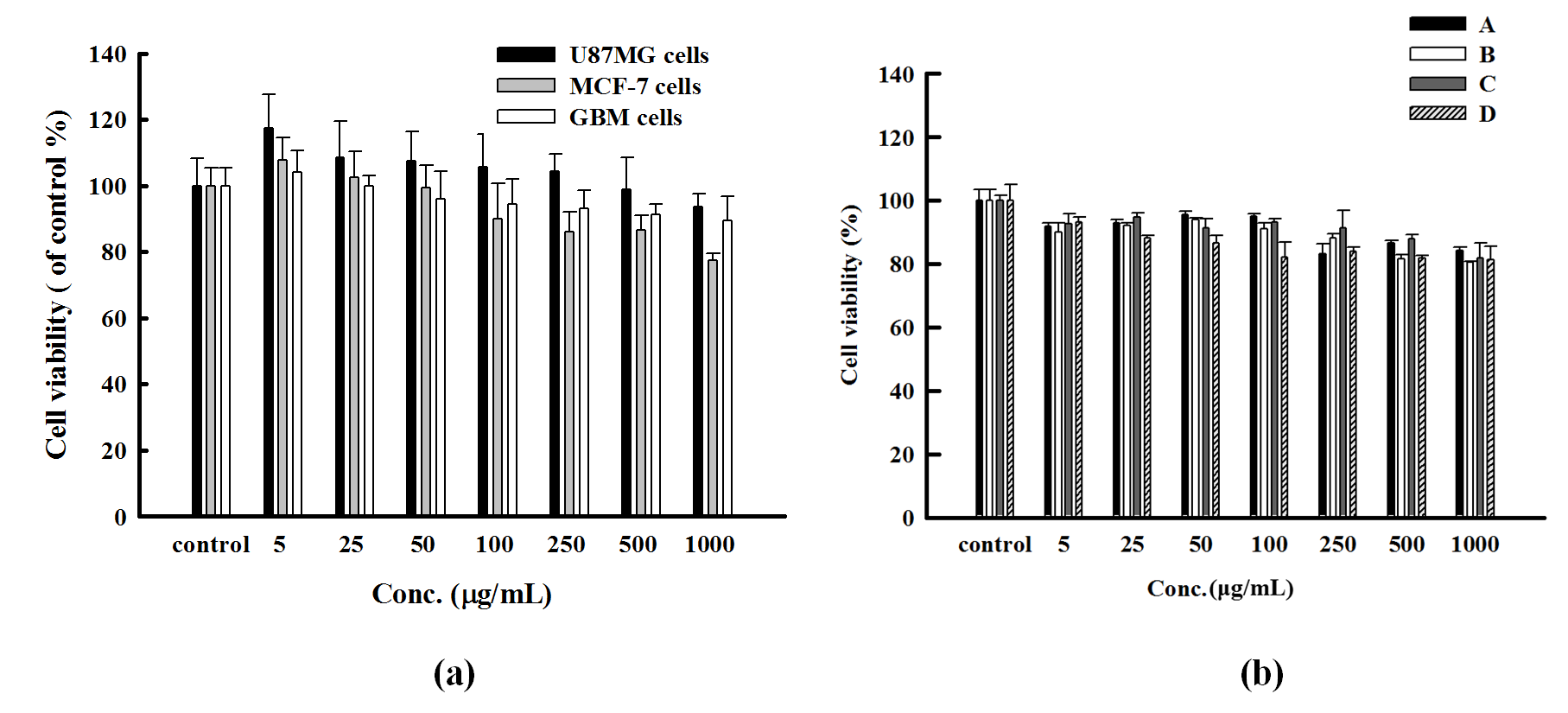

Supplement: Figure S7 — Pernicious biological properties test for the nanoparticle-loaded chitosan spheres. (a) The cytotoxicity of iron oxide nanoparticles-loaded chitosan spheres (5.4±1.7 nanometers of iron oxide nanoparticles (with 5% NaOH) inside chitosan spheres) with three cell lines. (b) The MTT assay data of various iron oxide nanoparticles loaded chitosan spheres (MCF-7 cells). Sample A: 5.4±1.7 nanometers of iron oxide nanoparticles (with 5% NaOH) inside chitosan spheres. Sample B: 4.4±0.9 nanometers of iron oxide nanoparticles (with 20% NaOH) inside chitosan spheres. Sample C: 3.8±0.9 nanometers of iron oxide nanoparticles (with 30% NaOH) inside chitosan spheres. Sample D: 2.5±0.2 nanometers of iron oxide nanoparticles (with 50% NaOH) inside chitosan spheres. (TIF) [file pone.0049329.s007.tif]
